# Interspecies protein substitution to investigate the role of the lyssavirus glycoprotein

**DOI:** 10.1099/vir.0.048827-0

**Published:** 2013-02

**Authors:** Denise A. Marston, Lorraine M. McElhinney, Ashley C. Banyard, Daniel L. Horton, Alejandro Núñez, Martin L. Koser, Matthias J. Schnell, Anthony R. Fooks

**Affiliations:** 1 Wildlife Zoonoses and Vector-borne Diseases Research Group, Animal Health and Veterinary Laboratories Agency, New Haw, Addlestone, Surrey, KT15 3NB, UK; 2 University of Liverpool, National Consortium for Zoonosis Research, Leahurst, Neston, South Wirral, CH64 7TE, UK; 3 Pathology Unit, Animal Health and Veterinary Laboratories Agency, New Haw, Addlestone, Surrey, KT15 3NB, UK; 4 Department of Microbiology and Immunology, Jefferson Vaccine Center, Thomas Jefferson University, Philadelphia, PA, USA

## Abstract

European bat lyssaviruses type 1 (EBLV-1) and type 2 (EBLV-2) circulate within bat populations throughout Europe and are capable of causing disease indistinguishable from that caused by classical rabies virus (RABV). However, the determinants of viral fitness and pathogenicity are poorly understood. Full-length genome clones based on the highly attenuated, non-neuroinvasive, RABV vaccine strain (SAD-B19) were constructed with the glycoprotein (G) of either SAD-B19 (SN), of EBLV-1 (SN-1) or EBLV-2 (SN-2). *In vitro* characterization of SN-1 and SN-2 in comparison to wild-type EBLVs demonstrated that the substitution of G affected the final virus titre and antigenicity. *In vivo*, following peripheral infection with a high viral dose (10^4^ f.f.u.), animals infected with SN-1 had reduced survivorship relative to infection with SN, resulting in survivorship similar to animals infected with EBLV-1. The histopathological changes and antigen distribution observed for SN-1 were more representative of those observed with SN than with EBLV-1. EBLV-2 was unable to achieve a titre equivalent to that of the other viruses. Therefore, a reduced-dose experiment (10^3^ f.f.u.) was undertaken *in vivo* to compare EBLV-2 and SN-2, which resulted in 100 % survivorship for all recombinant viruses (SN, SN-1 and SN-2) while clinical disease developed in mice infected with the EBLVs. These data indicate that interspecies replacement of G has an effect on virus titre *in vitro*, probably as a result of suboptimal G–matrix protein interactions, and influences the survival outcome following a peripheral challenge with a high virus titre in mice.

## Introduction

The lyssaviruses constitute an important group of zoonotic viral pathogens that continue to threaten both human and animal health globally. These viruses contain non-segmented negative-strand RNA genomes and are classified within the order *Mononegavirales*, family *Rhabdoviridae*, genus *Lyssavirus*. The genus *Lyssavirus* is divided into 14 species, 12 classified (http://www.ictvonline.org/virusTaxonomy.asp; accessed 26 November 2012) and two awaiting classification. The majority of lyssavirus species have been detected in various bat species and as such are speculated to have originated in bats.

The most globally important virus within this genus is rabies virus (RABV). This virus has been largely eliminated throughout western Europe in both domestic and wild terrestrial carnivore species. However, a threat, albeit low, to both public and animal health exists through the presence of two bat lyssavirus species in European bat populations: European bat lyssavirus type 1 (EBLV-1) and type 2 (EBLV-2). Two distinct lineages of EBLV-1 have been defined (Amengual *et al.*, 1997). EBLV-1 is most commonly associated with *Eptesicus serotinus*, with >90 % of cases being associated with this species (Müller *et al.*, 2007). Serological surveillance has shown long-term survival after seroconversion within bat populations across Europe (Brookes *et al.*, 2005; Echevarría *et al.*, 2001; Harris *et al.*, 2009; Serra-Cobo *et al.*, 2002). Where clinical disease is seen, bats are often weak and unable to fly and display abnormal behaviour, including uncoordinated movements, spasms and occasionally paralysis.

In contrast to EBLV-1, there have been far fewer isolations of EBLV-2 (McElhinney *et al.*, 2012). EBLV-2 was first isolated in 1985 from a Swiss bat biologist who had been working with bats in Finland, Switzerland and Malaysia. The first isolation from a bat was in Switzerland from *Myotis daubentonii*, the species from which the majority of isolations have been reported across Europe. The UK has reported nine isolations of EBLV-2 in *M. daubentonii* (Horton *et al*., 2009) and one human case in 2002 (Fooks *et al.*, 2003). In the Netherlands, EBLV-2 has been isolated from *Myotis dasycneme*. EBLV-2 infection in bats also results in disease indistinguishable from rabies. Studies in the UK suggest a low-level persistence in the bat population, with a seroprevalence estimate between 1 and 4 % (Harris *et al.*, 2009), but transmission dynamics for EBLV-2, and other lyssaviruses in bats, are still unclear.

All lyssaviruses contain five genes encoding the following proteins: nucleoprotein (N), phosphoprotein (P), matrix protein (M), glycoprotein (G) and the viral RNA polymerase (L). Expression of these genes is tightly regulated by short UTRs positioned at the genome termini, as well as signal sequences present at gene boundaries (Marston *et al.*, 2007; Orbanz & Finke, 2010). RABV was the first virus to be rescued from a DNA genome copy (Schnell *et al.*, 1994). Since then, numerous other studies have detailed the application of reverse genetics to non-segmented negative-strand RNA viruses, including other members of the family *Rhabdoviridae* (Lawson *et al.*, 1995).

Reverse-genetics techniques have been developed for two lyssavirus species: RABV (Buchholz *et al.*, 1999; Finke & Conzelmann, 1999; Schnell *et al.*, 1994) and EBLV-1 (Orbanz & Finke, 2010). The majority of studies have involved RABV and manipulations including insertion of novel transcription cassettes (Klingen *et al.*, 2008; Mebatsion *et al.*, 1996), tagging of viral proteins (Finke *et al.*, 2004; Klingen *et al.*, 2008), addition of extra copies of the G protein (Cenna *et al.*, 2008; Faber *et al.*, 2007), gene deletions (McKenna *et al.*, 2004), and often use as a vehicle to deliver foreign immunogens as a potential vaccine (Faber *et al.*, 2005b; Faul *et al.*, 2008, 2009; McGettigan *et al.*, 2003; Siler *et al.*, 2002). This last approach has been adapted recently to utilize an attenuated RABV expressing a filovirus G as a novel vaccine (Blaney *et al.*, 2011).

The lyssavirus G is required for virus entry into cells, by interacting with cell receptors and promoting virus and cell membrane fusion (Morimoto *et al.*, 2000). Changes in G can have a profound effect on the ability of viruses to infect and spread both *in vitro* and *in vivo* (Etessami *et al.*, 2000; Faber *et al.*, 2005a; Mebatsion *et al.*, 1996). Studies using RABV variants have shown an inverse correlation between virus titre in tissue culture and pathogenicity (Faber *et al.*, 2004; Morimoto *et al.*, 1999). The lyssavirus G is also the major antigen stimulating production of antibodies (Cox *et al.*, 1977). In addition to the ectodomain, the cytoplasmic domains of G also contribute to virus pathogenicity via cellular protein-binding sites (Préhaud *et al.*, 2010). A study investigating chimeric RABVs with the ectodomain and cytoplasmic domains of EBLV-1 and EBLV-2 was published recently (Genz *et al.*, 2012). This work showed that chimeric viruses were able to replicate successfully and cause clinical disease following intracranial (ic) inoculation. Here we assess the influence of G on pathogenicity, virus fitness and neuroinvasiveness using a recombinant attenuated RABV whose entire G has been replaced by EBLV-1- and EBLV-2-derived heterologous proteins.

## Results

### Rescue and titration of SN-1 and SN-2 recombinant viruses

We have rescued recombinant viruses containing G from EBLV-1 (SN-1) and -2 (SN-2) within an RABV backbone (SN) using RABV helper plasmids. SN-2 required seven passages to reach 100 % infectivity, SN and SN-1 required only two passages. Sequencing of the recombinant viruses was undertaken to confirm the appropriate EBLV G sequence was in frame with no errors and no changes due to cell passage (data not shown). All viruses were grown to 100 % infectivity and titrated. EBLV-2 grew to the lowest titre (5×10^4^ f.f.u. ml^−1^), 1 log_10_ lower than EBLV-1 (5×10^5^ f.f.u. ml^−1^). Both recombinant viruses have an increased titre by comparison with the EBLV equivalent (SN-1: 1.6×10^6^ f.f.u. ml^−1^ and SN-2: 4.16×10^5^ f.f.u. ml^−1^), although still lower than the SN virus (6.6×10^6^ f.f.u. ml^−1^).

### Measuring the antigenic effect of G

**Fig. 1.**
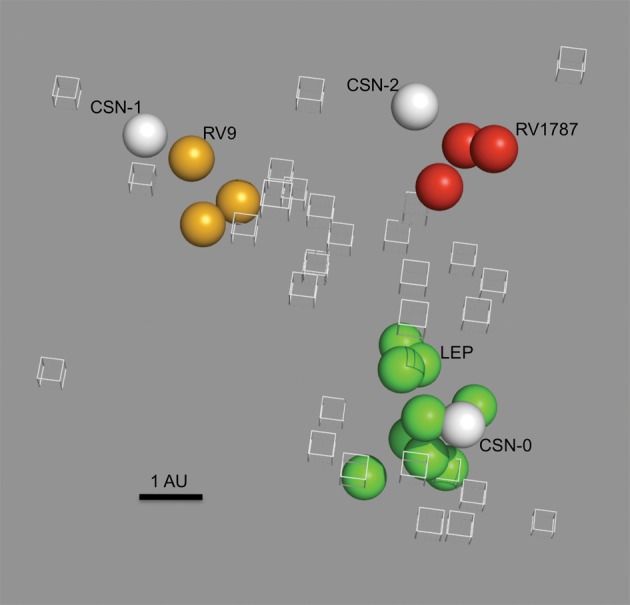
Three-dimensional antigenic map showing the antigenic relationship between recombinant viruses and wild-type viruses. Viruses (spheres) and sera (open boxes) are positioned such that the distance from each serum to each virus is determined by the neutralization titre. Antigenic cartography is used to position both sera and viruses relative to each other, so orientation of the map within the axes is free. Bar, 1 AU (antigenic unit), equivalent to a twofold dilution in antibody titre. Green spheres, RABV strains [Flury low egg passage (LEP) vaccine strain is labelled]; yellow spheres, EBLV-1 strains (RV9 is labelled); red spheres, EBLV-2 srains (RV1787 is labelled); labelled white spheres, recombinant viruses.

We hypothesized that the recombinant viruses SN-1 and SN-2 would be antigenically indistinguishable from EBLV-1 or EBLV-2 wild-type viruses, respectively, regardless of the backbone virus sequence. In order to test this hypothesis, the antigenic effect of G replacement was measured by comparing SN, SN-1 and SN-2 to wild-type viruses using antigenic cartography (Smith *et al.*, 2004; Horton *et al.*, 2010). The resulting antigenic map (Fig. 1) demonstrates that SN-1 clusters antigenically with EBLV-1 isolates, SN-2 clusters antigenically with EBLV-2 isolates, and the backbone virus, SN, clusters with other RABV isolates. Because this methodology uses polyclonal rabbit antisera in a neutralizing assay, it confirms that G is the dominant target for neutralizing antibodies and that SN-1 and SN-2 present antigenically as EBLV-1 and EBLV-2, respectively.

### *In vitro* single- and multi-step growth kinetics

**Fig. 2.**
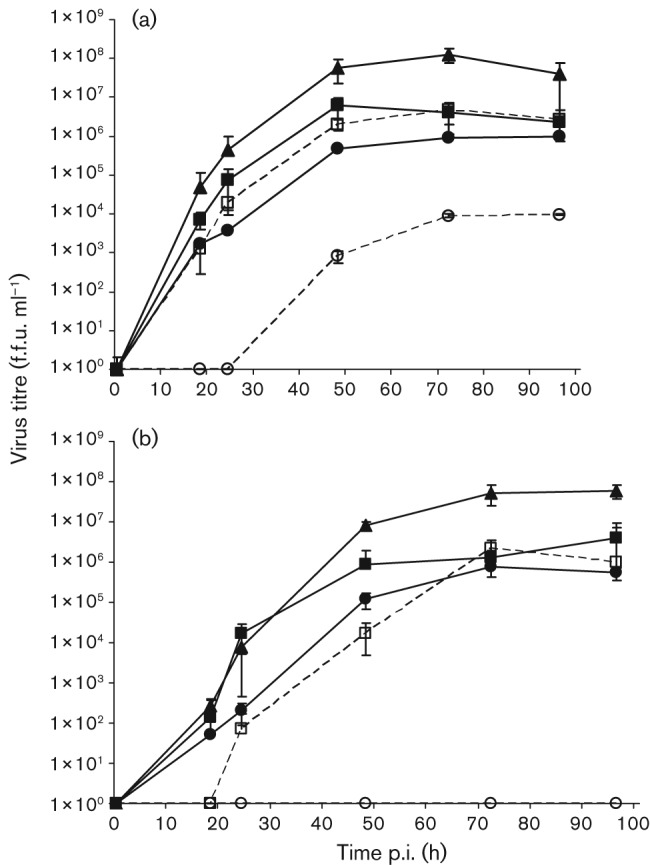
Single-step (a) and multi-step (b) growth curves of recombinant viruses SN-1 and SN-2 with EBLV-1 and EBLV-2 and SN backbone. Cells were infected at an m.o.i. of 1 for the single-step or 0.01 for the multi-step growth curve. Aliquots of the cultured supernatants were collected at the indicated time points and virus titres were determined in triplicate on BHK cells. For SN (▴), SN-1 (▪) and EBLV-1 (□), both time courses were undertaken twice and the mean of the combined results are displayed. For SN-2 (•) and EBLV-2 (○), the time courses were undertaken once.

Single- and multi-step growth curves were undertaken on baby hamster kidney (BHK) cells to determine the growth characteristics of the recombinant viruses (Fig. 2a, b). All viruses, with the exception of EBLV-2, were detected in the supernatant within 18 h post-infection (p.i.) in the single-step growth curves (m.o.i. 1; Fig. 2a). The titre of SN-1 virus detected during the first 24 h was between the titres for SN and EBLV-1, however, the final titre for SN-1 (2.29×10^6^ f.f.u. ml^−1^) was comparable to EBLV-1 (2.69×10^6^ f.f.u. ml^−1^), 17-fold lower than that of SN (3.94×10^7^ f.f.u. ml^−1^) (*P*<0.001). EBLV-2 was not detectable in the supernatant until 24 h, reaching an end point titre of 9.67×10^3^ f.f.u. ml^−1^ after 96 h. Conversely, the SN-2 virus was detectable after 18 h (1.75×10^3^ f.f.u. ml^−1^) reaching an end point titre of 1×10^6^ f.f.u. ml^−1^, over 2 logs higher than EBLV-2 (*P*<0.001), although a log lower than SN (*P* = 0.002). The multi-step growth curves (m.o.i. 0.01) followed the same trends as the single-step growth curves, however, EBLV-2 failed to establish a detectable infection, although staining of the monolayer at the end of the experiment indicated presence of the virus in a proportion of the cells. This failure to initiate an infection was observed in three separate experiments. Infection with EBLV-1, although established, was delayed by 18 h in comparison to the one-step growth kinetics data. Where a comparison can be made, there was at least 1.5 logs (3 logs for EBLV-1) difference between the equivalent titres when comparing the two growth curves at 18 h (Fig. 2).

### Pathogenicity and neuroinvasiveness of recombinant viruses in mice

**Fig. 3.**
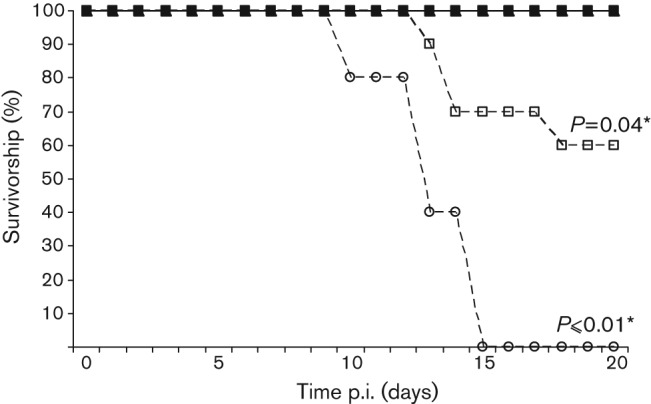
Pathogenicity of SN (▴), SN-1 (▪), SN-2 (•), EBLV-1 (□) and EBLV-2 (○) in mice. Groups of CD1 mice were injected via fp with 1×10^3^ f.f.u. of each virus. Mice were observed twice daily and euthanized when neurological signs were observed. The experiment was terminated after 28 days, but no clinical signs were observed past day 18. *Significant difference in survivorship as determined by Fisher’s exact test (CI = 90 %).

To determine the effect of G on the ability of the virus to cause clinical disease, CD1 mice were inoculated with 100 f.f.u. ic, and 1000 f.f.u. peripherally via the footpad (fp). The animals were monitored twice daily for clinical signs of rabies. Mice infected via the ic route all developed clinical disease consistent with lyssavirus infection and were euthanized at humane clinical end points, demonstrating that all the viruses were able to replicate successfully in the central nervous system (CNS) (data not shown). In contrast, 100 % of the mice survived the low dose (10^3^ f.f.u.) challenge with SN, SN-1 and SN-2 via a peripheral route (fp); only mice challenged with EBLV-1 and EBLV-2 developed clinical signs (Fig. 3). All sera from each recombinant virus fp group were tested for neutralizing antibodies using the fluorescent-antibody virus neutralization (FAVN) assay (Cliquet *et al.*, 1998) to confirm successful inoculation in the absence of clinical disease. All sera from SN and SN-2 and all but two sera from SN-1 groups had detectable antibody levels (data not shown).

**Fig. 4.**
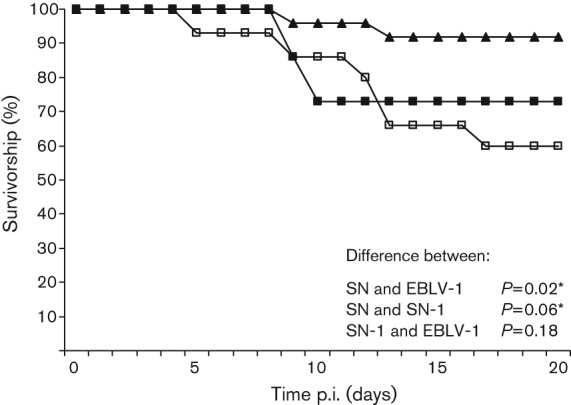
Pathogenicity of SN (▴), SN-1 (▪) and EBLV-1 (□) in mice. Groups of CD1 mice were injected via fp with 10^4^ f.f.u. of SN (*n* = 25), SN-1 (*n* = 30) and EBLV-1 (*n* = 15). Mice were observed twice daily and euthanized when neurological signs were observed. The experiment was terminated after 28 days, but no clinical signs were observed past day 17. *Significant difference in survivorship as determined by Fisher’s exact test (CI = 90 %).

A 50% mouse lethal dose (MLD_50_) challenge experiment (ic and fp) was undertaken with all viruses (except SN-2 which was unavailable at the time) to determine whether survival is dose-dependent (Fig. S1, available in JGV Online). An expected correlation between titre of the virus and survivorship was observed for all viruses inoculated via the ic route, with the EBLV-1 and EBLV-2 plots being indistinguishable from each other (Fig. S1a). The peripheral (fp) challenge results demonstrated that only the EBLV-2 had a dose-dependent effect, and clinical signs were observed for both EBLVs at a challenge as low as 10^1^ f.f.u. (Fig. S1b). In contrast, 100 % of animals survived when challenged with ≤10^3^ f.f.u. of SN and SN-1. Only when challenged with 10^4^ f.f.u. were clinical signs and a reduction in survivorship observed. A higher 10^5^ f.f.u. (neat virus) challenge was possible for SN, although the number of animals that developed clinical disease following inoculation with this higher dose only increased by one. The significance of the SN-1 survivorship graph with an inoculation dose of 10^4^ f.f.u. (neat virus) was unclear with small sample sizes (*n* = 5); therefore, a second experiment was carried out using 10^4^ f.f.u. EBLV-1, SN-1 and SN. EBLV-2 and SN-2 were not included as EBLV-2 did not achieve this titre. The results from the second experiment were comparable to the pilot MLD_50_ results (Fig. 4). Fisher’s exact test was used to determine the significance of these data, by combining results from all *in vivo* experiments performed with SN, SN-1 and EBLV-1 over the course of the investigation, using the same experimental design and conditions. The combined values were 60, 73 and 92 % survivorship for EBLV-1, SN-1 and SN, respectively. Statistically, the difference in survivorship between SN and EBLV-1 was significant (*P* = 0.02) and survivorship between SN and SN-1 was significant at the 90 % confidence interval (CI) level (*P* = 0.06). Importantly, there was no significant difference between survivorship following infection with EBLV-1 and SN-1 (*P* = 0.18) (Fig. 4).

### Histopathology and immunohistochemical distribution of viral antigen

**Fig. 5.**
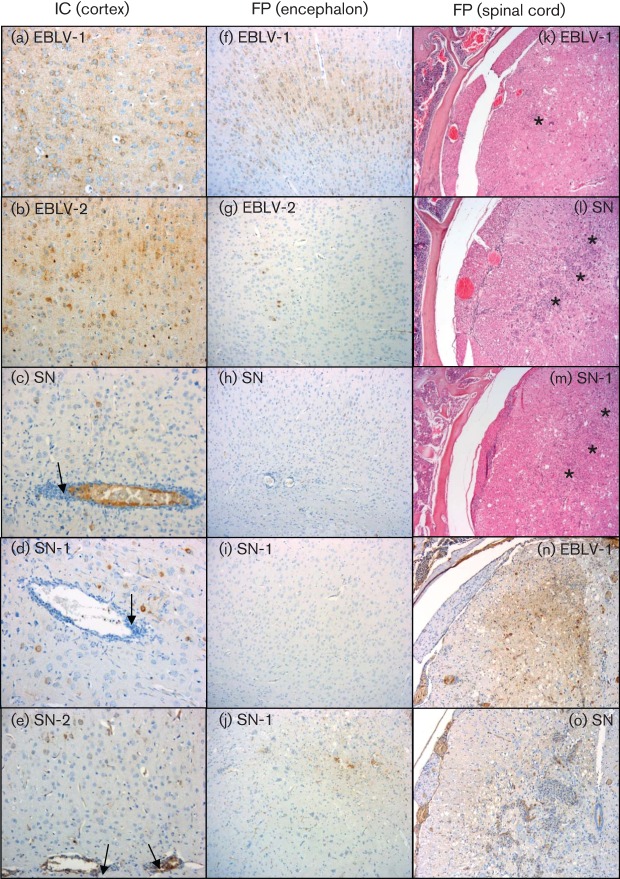
Histopathology and immunohistochemical demonstration of lyssavirus antigen. (a–e) Detection of viral antigen in cortex of ic-inoculated mice. EBLV-1 (a) and EBLV-2 (b) display abundant intraneuronal labelling (brown), while SN (c), SN-1 (d) and SN-2 (e) infection resulted in fewer infected neurons but more severe inflammatory changes including numerous perivascular cuffs (arrows). (f–j) Demonstration of lyssavirus antigen in cortex of peripherally (fp) inoculated animals. EBLV-1-challenged animals displayed abundant positive neurons (f); a smaller number in EBLV-2-challenged mice (g) and neurons were negative for SN (h) and SN-1 (i). Viral antigen demonstration in the vestibular nuclei of SN-1 peripherally challenged mice (j). (k–o) Histopathological changes in the thoracic spinal cord (DRG). Mild poliomyelitis was observed following EBLV-1 inoculation (k), while more severe changes were observed after (k) EBLV-1, (l) SN and (m) SN-1 infection with marked gliosis and glial nodule formation (*). (n, o) Viral antigen detection in thoracic spinal cord of peripherally challenged mice. Abundant immunolabelled cells after EBLV-1 infection (n) contrast with the very limited immunolabelling observed with SN (o).

Following ic inoculation, all viruses caused clinical disease. Histopathologically, there was evidence of viral encephalitis with all viruses, with qualitative differences in the severity of the inflammatory changes in the brain depending on the virus used. Presence of lyssavirus antigen in neurons in the brain was observed in all animals examined, with antigen evident throughout the encephalon, independent of the virus inoculated (data not shown). Clear antigen staining was observed following EBLV-1 and EBLV-2 infection (Fig. 5a, b, respectively), while antigen was detected to a lesser degree in mice infected with the recombinant viruses (SN, SN-1 and SN-2) (Fig. 5c–e). Antigen-specific immunolabelling in neurons was stronger for the EBLVs than for the recombinant viruses. In contrast, inflammatory changes were more severe for mice infected with the recombinant viruses. Inflammatory responses ranged from moderate to severe, with perivascular cuffing being evident in the recombinant virus-infected brains (Fig. 5c–e). There were no significant histopathological changes observed in the spinal cord sections in any of the animals, but virus antigen was detected in the grey matter of the spinal cord at cervical, thoracic and lumbar levels and in the neurons of dorsal root ganglia (DRG) with all viruses (data not shown).

All mice that reached a clinical end point following peripheral inoculation (fp) displayed changes in the encephalon and spinal cord consistent with viral encephalomyelitis (no animals inoculated with SN-2 virus displayed clinical signs 28 days post-inoculation, therefore no analysis was undertaken). Encephalitis was mild in EBLV-1-inoculated animals, mild to moderate in EBLV-2- and moderate to severe for SN- and SN-1-challenged mice (data not shown). Changes in specific nuclei (e.g. the vestibular nuclei) were more severe in SN- and SN-1-inoculated animals than in EBLV-1- or EBLV-2-infected mice. Intraneuronal immunolabelling against lyssavirus antigen was observed in all animals examined following a peripheral challenge. EBLV-1 showed the higher number of immunolabelled neurons, followed by EBLV-2 (Fig. 5f, g, respectively). The number of immunolabelled neurons was higher in both EBLVs than in SN- and SN-1-challenged animals, where sometimes immunolabelling was only restricted to cellular debris (Fig. 5f–j). The pons and medulla had high numbers of immunolabelled neurons with all four viruses. In the spinal cord, all clinically affected animals developed myelitis and ganglionitis. The changes were more severe in the thoracic sections of the spinal cord. The presence of perivascular cuffing was variable and not prominent. The severity of pathological changes observed in the spinal cord following peripheral inoculation was greater with SN and SN-1 (Fig. 5l, m, respectively), with prominent glial nodule formation. EBLV-1 and EBLV-2 elicited milder, diffuse microglial activation that was slightly more marked in EBLV-2-infected mice, with evident lymphocyte infiltration (Fig. 5k). Intraneuronal immunolabelling against lyssavirus antigen was observed in the spinal cord (Fig. 5n) and ganglion neurons of most sections in both EBLV-1 and EBLV-2. Similar to the observations in the brain, there were almost no immunolabelled cells in the spinal cord of SN- and SN-1-inoculated animals, in contrast with the EBLVs (Fig. 5o). No specific immunolabelling was observed in any of the peripheral tissues examined, including salivary gland, in any of the mice regardless of the route of inoculation or virus isolate used.

## Discussion

Generation of recombinant RABVs containing heterologous G proteins has shown that RABV can tolerate interspecies G substitution. The SN full-length RABV clone was manipulated such that G was replaced with that from either EBLV-1 (SN-1) or EBLV-2 (SN-2). These recombinant RABVs were able to grow in both *in vitro* and *in vivo* models, confirming their ability to replicate successfully and spread between cells. Antigenic cartography was used to measure the antigenic effect of replacing G in SN-1 and SN-2 by placing them on an antigenic map alongside other RABV, EBLV-1 and EBLV-2 viruses. SN-1 and SN-2 were antigenically indistinguishable from their wild-type counterparts (EBLV-1 and EBLV-2, respectively), distinct from SN (RABV), confirming that G is the dominant antigenic component of lyssaviruses, as determined by neutralizing antibodies (Fig. 1). These analyses also demonstrated the potential to use reverse genetics and antigenic cartography to test hypotheses regarding the antigenic dominance of individual epitopes within the G.

Replacing the entire G ORF (as opposed to just the ectodomain) has enabled investigation of the function of the EBLV G in an RABV backbone. Previous work with RABVs using a similar system has shown that replacing SN G with pathogenic RABV G has no significant effect on the SN virus (Morimoto *et al.*, 2000). Amino acid divergence between G may suggest that proteins from each species have evolved to interact optimally, therefore with only 70.3 % (CVS : EBLV-1) and 74.3 % (CVS : EBLV-2) amino acid identity, cross-species protein interactions could be affected. Indeed, the *in vitro* results indicate that SN-1 and SN-2 decrease in fitness compared with the homologous SN virus in both single- and multi-step time-course experiments (Fig. 2). The reduction in virus titre was evident from not only the peak titre reached by each virus, but also the titre at each time point. However, it is important to analyse these data alongside the growth kinetics for each of the respective EBLVs. Both SN-1 and SN-2 improved their viral growth curve kinetics in comparison to the EBLVs, indicating that the EBLV G is able to interact with heterologous RABV M protein during encapsidation and budding. Moreover, this suggests that the EBLV G is not the dominant protein influencing virus titre in EBLVs, as the recombinant viruses grow to equivalent (SN-1), or higher (SN-2) titres than EBLV-1 and EBLV-2, respectively, despite any disadvantage of heterologous protein interactions. This observation would indicate that the low titres observed for EBLVs are not a result of G-dependent processes such as receptor binding or entry, rather, of G-independent steps of virus replication. A previous study investigating the role of the ectodomain and transmembrane regions of EBLVs in an RABV backbone did not find significant differences in the growth kinetics of the chimeric viruses in comparison to the RABV backbone, suggesting that the cytoplasmic region of G is influencing the reduction in virus titre observed in the results described here (Genz *et al.*, 2012). The study postulated that the cytoplasmic domain may influence virus replication as a result of impartial or incomplete interactions with the ribonucleoprotein (RNP) or a heterologous M protein, which in turn may lead to inefficient virus release.

SN is a recombinant attenuated virus derived from SAD-B19 (GenBank accession no. M31046), which has been well characterized and studied, particularly in a murine model where all animals survive a peripheral challenge (Faber *et al.*, 2004). The importance of G for neuroinvasion was investigated by replacing the G gene of SN, with a G gene from two neuroinvasive lyssaviruses and inoculating both via ic and peripheral (fp) routes. The pathogenic ability of the recombinant and backbone viruses in relation to the wild-type viruses was confirmed by ic inoculation. However, to study the effects of G via a route mimicking natural exposure, a peripheral challenge was also undertaken. Where possible, an MLD_50_ was undertaken to determine: (i) whether altering the virus dose results in dose-dependent survivorship curves; and (ii) the optimal quantity of virus inocula to give a comparable dataset. The SN, SN-1 and SN-2 Kaplan–Meier survival curves show that there is a threshold, under which all animals survive a peripheral challenge, and over which one or two animals may succumb (Fig. S1), rather than a dose-dependent response. Because SN is an attenuated, cell-adapted virus, inoculation of higher doses of SN, SN-1 and SN-2 was possible. For SN, a high dose (higher than achieved for any wild-type EBLV) was required to observe any clinical signs, in one (20 %) of the animals. In contrast, the EBLVs are not cell-culture-adapted and are pathogenic at lower doses. The EBLV titration resulted in animals succumbing to as little as 10 f.f.u. (2 log_10_ lower than the rescued viruses), a result that may reflect the selective pressure on wild-type viruses to minimize replication to evade the immune system while increasing pathogenicity and neuroinvasiveness. By swapping G between laboratory-adapted and wild-type lyssavirus isolates, we have addressed the significance of G in neuroinvasiveness and pathogenicity. All viruses were compared at 10^3^ f.f.u. in a peripheral *in vivo* challenge (Fig. 3). At this dose, there was no increased neuroinvasiveness observed in SN-1 and SN-2 over SN. Only the EBLVs resulted in clinical disease significant in comparison to the recombinant viruses (*P* = 0.01 EBLV-2 and *P* = 0.04 EBLV-1). The SN-1 peripheral challenge study at 10^4^ f.f.u. resulted in 73 % survival, which is not significantly different from EBLV-1 (*P* = 0.18) (Fig. 4). In contrast, the difference observed between the SN and SN-1 Kaplan–Meier plots is significant (at the 90 % CI level; *P* = 0.06), indicating the G substitution has an effect on the neuroinvasive properties of the SN virus. Regrettably, SN-2 could not be compared at this high titre. The involvement of other viral proteins remains unclear, but M has previously been shown to support budding and therefore play a role in virus cell-to-cell spread (Mebatsion *et al.*, 1999; Pulmanausahakul *et al.*, 2008). Thus it appears more likely that a combination of differences across the viral genome, rather than a single gene, contribute to the attenuated phenotype of the SAD-B19 strain.

Histopathological analysis was undertaken on the clinical animals from both the ic- and fp-inoculated groups. In the ic-inoculated mice, all viruses spread from the brain into the spinal cord and also to the neuronal bodies of the sensory neurons (DRG) in the peripheral nervous system with equal ability. For the fp-inoculated mice, SN and SN-1 resulted in more severe inflammatory changes in the brain and appeared to display fewer immunolabelled neurons than EBLV-1. This increased inflammatory response may be partially responsible for limiting the spread of the virus, or just the consequence of neuronal destruction (Hicks *et al.*, 2009). The higher survival rate of the mice inoculated peripherally with the recombinant viruses over the wild-type viruses supports this observation, suggesting that there is an inverse correlation between the neuroinvasiveness of the virus and the inflammation observed. For both routes, despite the G present, the histopathological changes and viral antigen distribution observed for SN-1 and SN-2 were more representative of those seen with the SN virus than that seen with the EBLVs. This indicates that the host response is more heavily influenced by other viral proteins important in the development of the histological lesions observed.

The manipulation of an RABV backbone virus (SN) to rescue recombinant viruses with complete G from two different lyssavirus species has demonstrated successfully that G is the dominant target for neutralizing antibodies. Furthermore, we have shown that interspecies G substitution reduces the virus titre in comparison with SN. Future work investigating the cytoplasmic domain of G may further define restrictions to interactions between G and M and/or RNP. Our observation that SN-2 end titre increased significantly (*P*<0.001) in comparison to EBLV-2 indicates that G-independent factors are responsible for the low titre levels observed for EBLV-2. The presence of G from a neuroinvasive virus in a non-neuroinvasive backbone appears to help the virus reach the CNS in the case of SN-1, albeit only at a high virus titre. The histopathological changes and viral antigen distribution were more representative of those seen with the SN virus than that of the neuroinvasive EBLVs. The constructs will be manipulated further to identify specific regions of G that are responsible for the neutralizing antibodies and cell entry. Furthermore, recombinant EBLV-1 and EBLV-2 backbones are being constructed to investigate homologous/heterologous protein interactions.

## Methods

### 

#### Cells and viruses.

BSR-T7 cells (BHK-derived cells that stably express T7 RNA polymerase) and BHK cells were grown in Dulbecco’s modified Eagle’s medium (DMEM; Invitrogen) supplemented with 10 % FBS, 100 units penicillin ml^−1^, 100 µg streptomycin ml^−1^ and 25 units mycostatin ml^−1^ (Invitrogen). BSR-T7 cells were grown in the presence of 1 mg G418 every third passage as described previously (Buchholz *et al.*, 1999) to maintain the T7 expression. N2a cells (a mouse neuroblastoma cell line) were grown in RPMI medium (Invitrogen) supplemented with 10 % FBS, 100 units penicillin ml^−1^, 100 µg streptomycin ml^−1^ and 25 units mycostatin ml^−1^.

EBLV-1 strain RV9 (GenBank accession no. EF157976) was originally isolated from a serotine bat (*Eptesicus serotinus*) as described previously (Marston *et al.*, 2007). EBLV-2 strain RV1787 was isolated from a Daubenton’s bat (*Myotis daubentonii*) in the UK (Fooks *et al.*, 2004). Viruses were propagated from original brain homogenate in N2a cells. Previously rescued SN virus (Schnell *et al.*, 1994) was cultured in BHK cells to produce a virus stock (P2). Recombinant viruses were also propagated in BHK cells.

#### Construction of recombinant cDNA clones.

The SN strain of RABV was derived from the SAD B19 cDNA clone as described previously (Faber *et al.*, 2004; McGettigan *et al.*, 2001; Morimoto *et al.*, 2000; Pulmanausahakul *et al.*, 2008). Glycoproteins (Gs) from EBLV-1 and EBLV-2 were amplified using Elongase (Invitrogen) and the following primers: EBLV1GEcoRVF (5′-TCAGATATCATGTTACTCTCTAC-3′) and EBLV1GNheIR (5′-AAGGCTAGCTTATGACTCACC-3′); EBLV2GEcoRVF (5′-GATCTCGATATCATGCCATTCC-3′) and EBLV2GNheIR (5′-AGCAAGGCTAGCTTAAGACTG-3′). Restriction sites used for cloning are underlined. The full-length SN was prepared by digestion with *Sma*I and *Nhe*I and the *Eco*RV/*Nhe*I-prepared G ORFs were cloned directionally. The recombinant constructs were designated SN-1 (EBLV-1 G) and SN-2 (EBLV-2 G). Resulting recombinant clones were sequenced to confirm the presence of the correct G gene sequence.

#### Virus rescue and *in vitro* quantification.

Viruses were rescued as described previously (Schnell *et al.*, 1994). Briefly, BSR-T7 cells were transfected with 5 µg full-length genome cDNA in addition to plasmids encoding the RABV N, P, L and G proteins using a Ca_2_PO_4_ transfection kit (Stratagene). Supernatants were transferred onto fresh BSR-T7 cells 3 days post-transfection, and infectious virus was detected 3 days later by immunostaining with an anti-lyssavirus nucleocapsid (N) protein-specific FITC-labelled antibody (Fujirebio). Supernatant from a positive well was used to generate virus stocks in BHK cells. Sequencing of final passage virus stocks confirmed the presence of heterologous Gs in the recombinant viruses.

Quantification of virus titre was performed *in vitro* by titration in BHK cells in triplicate as described previously (Faber *et al.*, 2004). At 48 h p.i. cells were fixed in 80 % acetone (Sigma) and stained with FITC-labelled RABV N-specific antibody (Fujirebio). Fluorescent foci were counted and virus titres were calculated as f.f.u.

#### Antigenic cartography.

Antigenic relationships between the recombinant viruses and wild-type viruses were quantified and visualized, using techniques described previously (Smith *et al*., 2004; Horton *et al*., 2010). Briefly, modified FAVN tests, using a fixed quantity of stock virus (100 TCID_50_), were used to measure the ability of a panel of 30 hyperimmune rabbit sera to neutralize each virus (RABV, *n* = 9; EBLV-2, *n* = 3; EBLV-1, *n* = 3) (Table S1). An antigenic map was generated using multi-dimensional scaling, with target distances derived from the neutralization titres. Multiple random-restart optimizations were used to minimize sum-squared error between target distance and distance on the map, and the maps with lowest sum-squared error were ranked and compared quantitatively for self-consistency. The maps were visualized in three dimensions using Pymol (DeLano Scientific LLC).

#### Single- and multi-step growth curves.

BHK cells were grown for 24 h and monolayers were infected at an m.o.i. of 1 for single-step growth curves and an m.o.i. of 0.01 for multi-step growth curves. After 1 h incubation at 37 °C, the inoculum was removed, the cells were washed three times with PBS, growth medium was added and the cells were incubated at 37 °C. Following infection, 100 µl tissue-culture supernatant was removed at set time points and immediately frozen at −80 °C. The frozen time-point aliquots were defrosted and titrated in triplicate on BHK cells twice, to give a total of six observations for each virus. The log_10_ values of the final virus titres were taken and the means were compared using a Student’s *t*-test.

#### *In vivo* studies.

All *in vivo* work was undertaken in BSL3/SAPO4 containment at the Animal Health and Veterinary Laboratories Agency (AHVLA), following independent ethical review and it complied with the Animal Scientific Procedures Act 1986. Groups of 3–6-week-old CD1 mice were infected under anaesthesia via either the intracranial (ic) or the footpad (fp) route with 20 µl virus. Negative-control mice were inoculated with medium alone. Mice were observed for 28 days and clinical signs were scored using a scale of 0–5 (where 0, no effect; 1, hunched body/ruffled fur; 2, limb twitching; 3, hindquarter paralysis; 4, progressive paralysis; 5, terminal recumbency/death). Mice were euthanized at clinical score 3. Kaplan–Meier survival curves and Fisher’s exact test were used to analyse the survivorship rates observed in the *in vivo* experiments. Where data from multiple experiments were used, uniformity in experimental design and execution was ensured.

#### Histopathology and immunohistochemistry.

Brains from mice with a clinical score of 3 were extracted and corporal cavities were opened prior to immersion in 10 % buffered formalin for a minimum period of 5 days. Coronal sections of the encephalon at six different levels were prepared as described previously (Hicks *et al.*, 2009). Cross sections of spine and spinal cord were prepared from the lumbar, thoracic and cervical regions after decalcification in EDTA (Sigma) for 1 week. Samples from salivary gland, mandibular and popliteal lymph nodes and spleen were dissected from the carcasses. All these tissues were routinely processed and embedded in paraffin wax. Sections 4 µm thick were either stained with haematoxylin (Surgipath) and eosin (VWR) for histopathological examination or used for the detection of RABV nucleocapsid by immunohistochemistry using the mAb HAM 5DF123B0 as primary antibody, as previously described (Hicks *et al.*, 2009).
